# Exploring the microbiota difference of bronchoalveolar lavage fluid between community-acquired pneumonia with or without COPD based on metagenomic sequencing: a retrospective study

**DOI:** 10.1186/s12890-024-03087-6

**Published:** 2024-06-12

**Authors:** Bingbing Wang, Min Tan, Wei Li, Qinghua Xu, Lianfeng Jin, Shuanshuan Xie, Changhui Wang

**Affiliations:** 1 Department of Respiratory Medicine, Shanghai Tenth People’s Hospital, School of Medicine, Tongji University, Shanghai, 200072 China; 2 Department of Geriatrics, Shanghai Tenth People’s Hospital, School of Medicine, Tongji University, Shanghai, 200072 China; 3 Vision Medicals Center for Infectious Disease, Guangzhou, Guangdong China

**Keywords:** Community acquired pneumonia, Chronic obstructive pulmonary disease, Bronchoalveolar lavage fluid, Metagenomic next-generation sequencing

## Abstract

**Background:**

Community-acquired pneumonia (CAP) patients with chronic obstructive pulmonary disease (COPD) have higher disease severity and mortality compared to those without COPD. However, deep investigation into microbiome distribution of lower respiratory tract of CAP with or without COPD was unknown.

**Methods:**

So we used metagenomic next generation sequencing (mNGS) to explore the microbiome differences between the two groups.

**Results:**

Thirty-six CAP without COPD and 11 CAP with COPD cases were retrieved. Bronchoalveolar lavage fluid (BALF) was collected and analyzed using untargeted mNGS and bioinformatic analysis. mNGS revealed that CAP with COPD group was abundant with Streptococcus, Prevotella, Bordetella at genus level and Cutibacterium acnes, Rothia mucilaginosa, Bordetella genomosp. 6 at species level. While CAP without COPD group was abundant with Ralstonia, Prevotella, Streptococcus at genus level and Ralstonia pickettii, Rothia mucilaginosa, Prevotella melaninogenica at species level. Meanwhile, both alpha and beta microbiome diversity was similar between groups. Linear discriminant analysis found that pa-raburkholderia, corynebacterium tuberculostearicum and staphylococcus hominis were more enriched in CAP without COPD group while the abundance of streptococcus intermedius, streptococcus constellatus, streptococcus milleri, fusarium was higher in CAP with COPD group.

**Conclusions:**

These findings revealed that concomitant COPD have an mild impact on lower airway microbiome of CAP patients.

## Background

Community acquired pneumonia (CAP) is one of the most frequent infectious causes of death [1–3]. Among those CAP patients who were older than 40 years, there are a specific proportion of them concomitant with chronic obstructive pulmonary disease (COPD) [4, 5]. COPD is a progressive pulmonary disorder characterized by persistent airflow limitation and respiratory symptoms, which is now the 3rd leading cause of death [6]. The diagnosis criteria of COPD consists of post-bronchodilator fixed ratio of FEV1/FVC < 70%, which means persistent airflow limitation [7]. A number of well designed large population based studies indicated that COPD is the strongest risk factor for CAP [8–10]. Meanwhile, several studies showed that CAP patients got worse disease severity in terms of Pneumonia Severity Index or CURB-65 when concomitant with COPD [11]. Previous studies have attempted to explain the complex etiology of CAP happened in COPD patients, mainly involving lung microbiome, host immunity and pathogen virulence. In general, unlike healthy lung microbiome which harbors low number but a high diversity of bacterium, COPD patients have a dysbiosis lung microbiome, characterized by low diversity [12, 13]. In clinic, considering CAP patients with COPD were at higher risk of deterioration, pulmonary physicians tend to choose a broader-spectrum antibiotics therapy. But the precise lower airway microbiome comparison of CAP with COPD and CAP without COPD is lacking. We would like to know whether there are any differences of the lower airway microbiome distribution between CAP with COPD and CAP without COPD by using metagenomic next generation sequencing (mNGS), an unbiased detection tool of bacteria, eukaryotes and DNA viruses at species level [14, 15].

## Methods

### Patient selection

Patients whose BALF (bronchoalveolar lavage fluid) had gone through mNGS in the Department of Respiratory Medicine of Shanghai Tenth People’s Hospital (Shanghai, China) between 2019 April to 2021 August were reviewed. Cases which met the diagnosis criteria of community-acquired pneumonia were included, in accordance with the American Thoracic Society/Infectious Diseases Society of America Community-Acquired Pneumonia Guideline [11]. Among those cases, the patients who matched these criteria: a) the presence of a post-bronchodilator FEV1/FVC < 0.7; b)acute deterioration of respiratory symptoms that need additional therapy such as antibiotics) were classified into CAP with COPD group. Otherwise, they would be classified into the CAP without COPD group. Lung carcinoma or immunocompromised patients were excluded.

### BALF collection and DNA extraction

Experienced clinicians guided the bronchoscopy to diseased region of the lungs referring to CT results. BALF was typically conducted using serial 20-ml fractions containing 60–100 ml of 0.9% NaCl. 60% of the volume of the lavage was extracted using a gentle syringe suction and transferred to sterile receptacles. Centrifuge the BALF samples at 1000 rpm for 10 min at 4 °C and supernatants and precipitation were separately frozen (− 80 °C) for further analysis. DNA was extracted from the precipitation using a QIAamp® UCP Pathogen DNA Kit (Qiagen) following the manufacturer provided protocol. Human DNA was removed using Benzonase (Qiagen) and Tween20 (Sigma) [16].

### Library preparation and sequencing

Ten nanograms DNA samples was used to construct library using Nextera XT DNA Library Prep Kit (Illumina, San Diego, CA) [17]. Library pools were then loaded onto an Illumina Nextseq 550Dx sequencer for 75 cycles of single-end sequencing to generate approximately 20 million reads for each library. Peripheral blood mononuclear cell samples with 10^5^ cells/mL from healthy donors was served as negative controls [17, 18]. DNA-free water also went through DNA extraction and mNGS analysis as a blank control group to assess the degree of background contamination.

### Bioinformatics analysis

Low quality reads, adapter contamination, and duplicate reads, reads shorter than 50 bp as well as low complexity reads with default parameters was removed using trimmomatic [19] and Kcomplexity [20]. Human sequence data were identified and excluded referring to a human reference genome (hg38) using Burrows-Wheeler Aligner software [21].

After alignment, multiple indicators were comprehensively evaluated to have the list of suspected microorganisms. Then, microbiota composition profiles were inferred from quality filtered forward reads using Kraken V.2.1.2 and Bracken V.2.6.2 with the k2_pluspf_20210517 database.

### Statistical analysis

The site by species counts and relative abundance tables were input into R-base V.4.1.0 for statistical analysis. Alpha diversity of the microbiota profile for each subject was assessed by group at the different level data using the Vegan package in R (version 2.5.7). Different groups were assessed using permutational multi-variate analysis of variance (PERMANOVA) and PCA stat with the Vegan package in R (version 2.5.7). The result of PCOA was stated by the function of dudi.pco in ade4 package in R (version 1.7.18). Associations of specific microbial species or genus with patient parameters were identified using the linear discriminant analysis effect size (LEfSe) [22]. LDA > 2 were considered significantly different.

## Results

A total of 36 CAP without COPD and 11 CAP with COPD cases were retrieved (Table 1). In the former group, 24 out of 36 were male while 10 out of 11 in latter group were male gender. As shown in the Table 1, the mean age, CURB 65 and serum biomarkers such as CRP, WBC, NEUT% and the comorbidity rate of hypertension and diabetes mellitus is not significantly different between groups. Body mass index (BMI) was significantly lower in CAP with COPD group (19.96 ± 2.11) compared with CAP without COPD group (23.05 ± 3.1). FEV1/pre was 58.53 ± 16.28 in CAP with COPD and 76.56 ± 20.16 in CAP without COPD. FEV1/FVC was 61.36 ± 9.64 in CAP with COPD and 80.29 ± 3.65 in CAP without COPD.

**Table 1:** Clinical characteristics of the study population

	CAP without COPD	CAP with COPD	*P* value
*N*	36	11	
Gender, male(%)	24(63.16%)	10(90.9%)	0.060
Age			
Range	50–85	57–77	
Mean ± sd	67.97 ± 8.79	67.08 ± 5.69	0.785
BMI			
Range	14.17–28.73	17.3–23.44	
Mean ± sd	23.05 ± 3.1	19.96 ± 2.11	0.002
Comorbidities			
Hypertension(%), *n*	14(38.89%)	2(18.18%)	0.366
Diabetes mellitus (%), *n*	6(16.67%)	3(27.27%)	0.730
CURB 65			
0–1	32(88.89%)	9(81.82%)	
≥ 2	4(11.11%)	2(18.18%)	0.921
WBC(× 10^9^/L)	6.86(5.86,10.61)	10.13(7.50,12.66)	0.090
NEUT%	73.55(63.98,77.60)	80.1(76.1,87.7)	0.297
CRP(mg/L)	41.89(3.14,139.56)	97.98(54.47,161.5)	0.167
FEV1/pre			
Range	43.6–117.6	20.2–80.6	
Mean ± sd	76.56 ± 20.16	58.53 ± 16.28	0.021
FEV1/FVC			
Range	73.79–87.43	36.61–69.72	
Mean ± sd	80.29 ± 3.65	61.36 ± 9.64	< 0.001

*CAP* community acquired pneumonia, *COPD* chronic obstructive pulmonary disease, *N* number, *sd* standard deviation, *BMI* body mass index, *WBC* white blood cell, *CRP* C-reactive protein, *FEV1* forced expiratory volume in 1 s, *FEV1/pre* FEV1 percent predicted, *FVC* forced vital capacity

Table 2 illustrates that the etiology of pneumonia of CAP without COPD group is 38.89% bacteria, 2.78% fungi, 5.55% atypical pathogen and 52.78% unknown, while that for CAP with COPD group is 45.45% bacteria and 54.55% unknown. The conventional cultures result of the BALF is also listed.

**Table 2:** Etiologic diagnosis of the pneumonia and result of conventional cultures of the bronchoalveolar lavage

	CAP without COPD(*n* = 36)	CAP with COPD(*n* = 11)
Etiology of pneumonia		
Bacteria(%), *n*	14(38.89%)	5(45.45%)
Fungi(%), *n*	1(2.78%)	0
Atypical pathogen(%), *n*	2(5.55%)	0
Virus(%), *n*	0	0
Unknown(%), *n*	19(52.78%)	6(54.55%)
Conventional cultures result of the BALF		
Candida albicans	6(16.67%)	1(9.09%)
Klebsiella pneumoniae	3(8.33%)	0
Acinetobacter baumannii	2(5.55%)	1(9.09%)
MRSE	1(2.78%)	1(9.09%)
Streptococcus pneumoniae	0	1(9.09%)
Viridans Streptococci	1(2.78%)	0
Stenotrophomonas maltophilia	1(2.78%)	0

*MRSE* methicillin-resistant Staphylococcus epidermidis

### Bacterial, viral and fungal composition

Deep metagenome sequencing can totally identified 1012 bacteria, 27 viruses and 18 fungi at genus level, and 3355 bacteria, 71 viruses and 22 fungi at specie level.

**Fig. 1:**
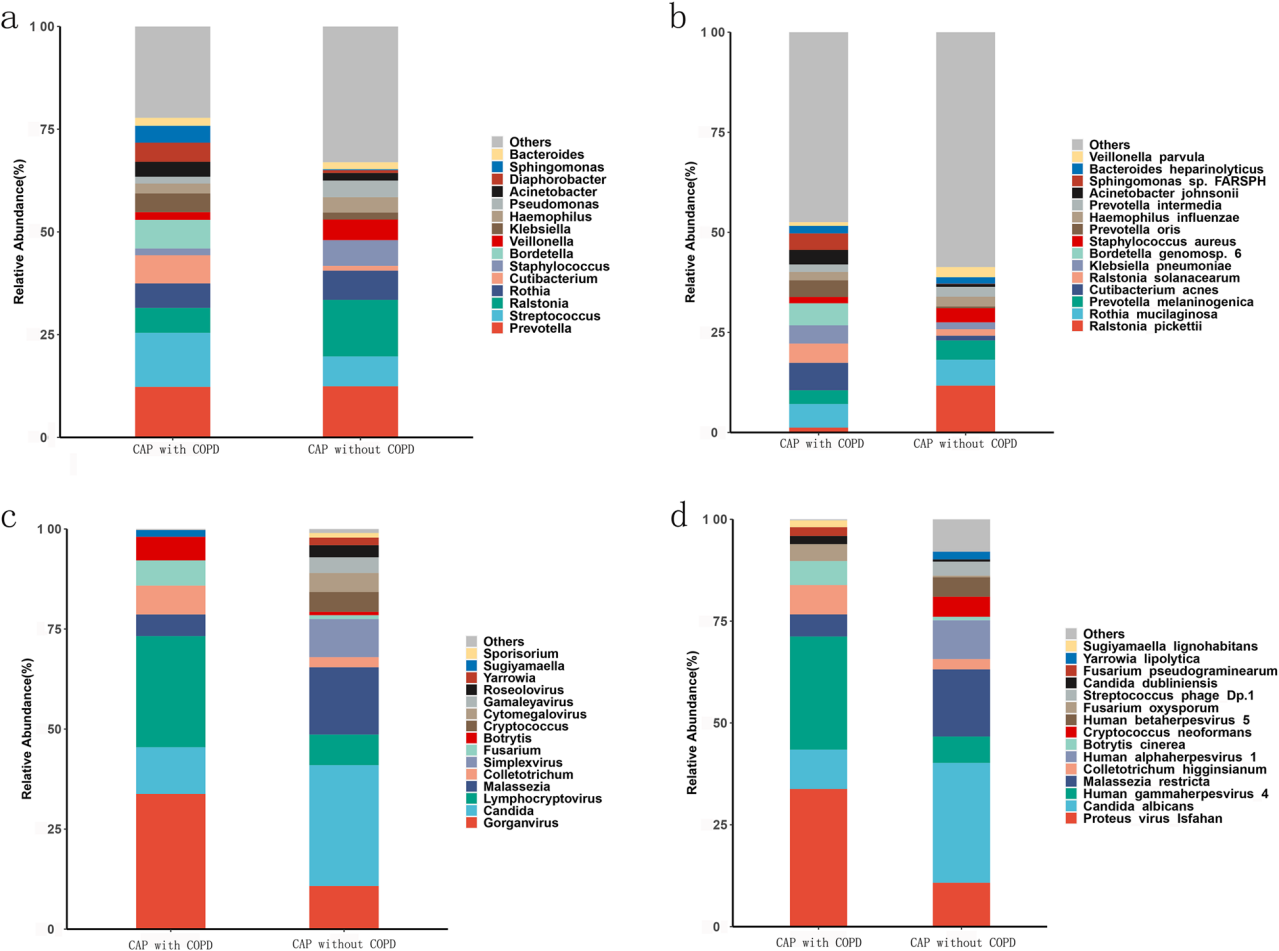
Relative abundance of top 15 microbiome taxa in CAP patients with COPD or without COPD. **a** Relative abundance of top 15 bacterial genera between CAP patients with COPD or without COPD. **b** Relative abundance of top 15 bacterial species between CAP patients with COPD or without COPD. **c** Relative abundance of top 15 fungal and viral genera between CAP patients with COPD or without COPD. **d** Relative abundance of top 15 fungal and viral species between CAP patients with COPD or without COPD

Figure 1a showed the top 15 bacteria genus and Fig. 1b showed the top 15 bacteria species respectively in CAP with COPD and CAP without COPD. In CAP with COPD group,the top 10 bacteria genus, from most abundance to least abundance, were Streptococcus, Prevotella, Bordetella, Cutibacterium, Ralstonia, Rothia, Klebsiella, Haemophilus, Veillonella, Staphylococcus. While in CAP group, from most abundance to least abundance, the top 10 bacteria genus were Ralstonia, Prevotella, Streptococcus, Rothia, Staphylococcus, Veillonella, Haemophilus, Cutibacterium, Bordetella etc. In CAP with COPD group, the top 10 bacteria species were Cutibacterium acnes, Rothia mucilaginosa, Bordetella genomosp. 6, etc. While in CAP without COPD group, the top 10 bacteria species were Ralstonia pickettii, Rothia mucilaginosa, Prevotella melaninogenica, etc.

The most common virus and fungus genus were Gorganvirus and Candida both in CAP with COPD and CAP without COPD group respectively (Fig. 1c). The most common virus and fungus species were Gorganvirus and Candida both in CAP with COPD and CAP without COPD group respectively (Fig. 1d).

### Microbiome diversity comparison

**Fig. 2:**
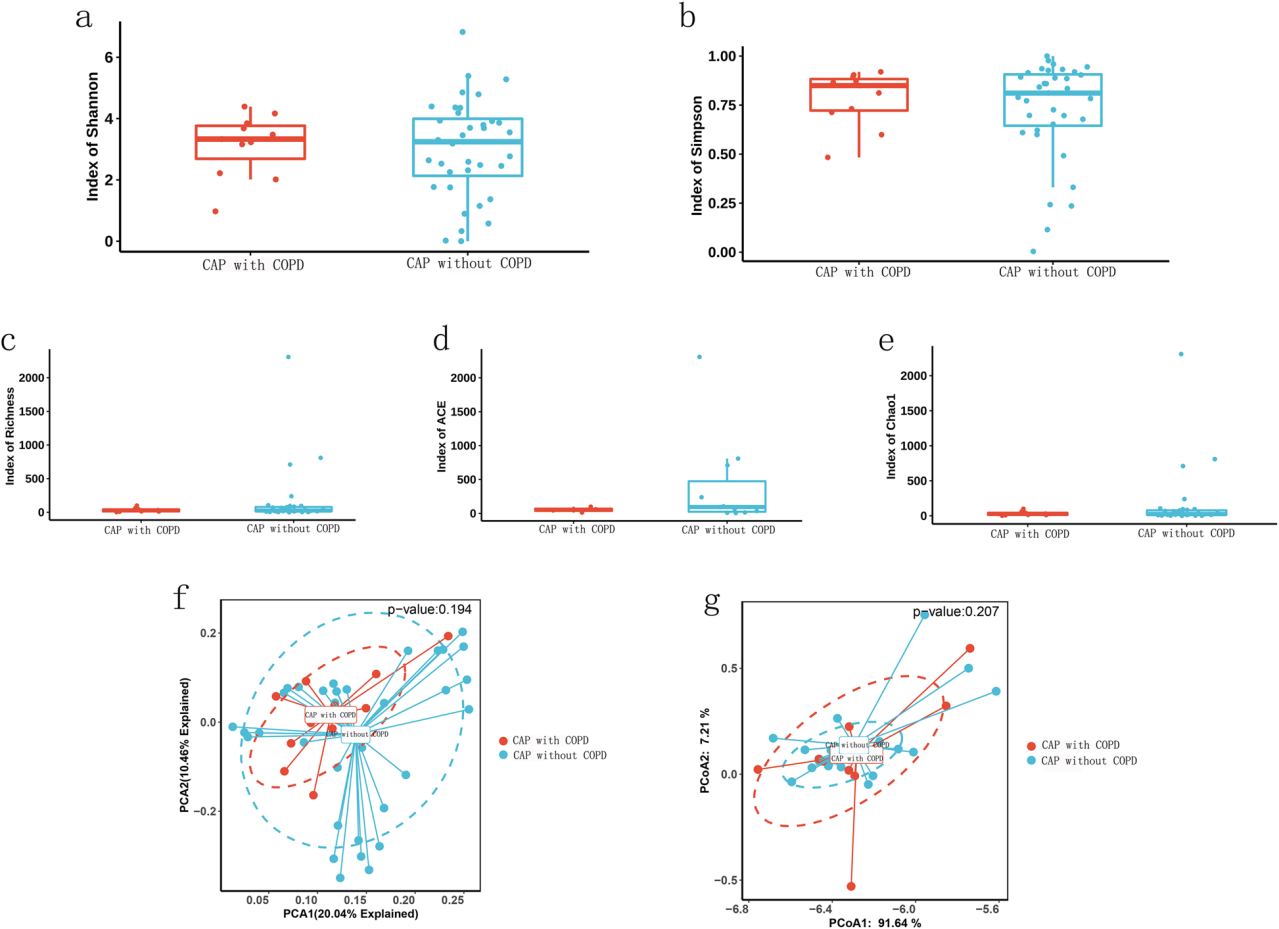
Alpha and beta diversity comparison between CAP patients with COPD or without COPD. (**a**) Shannon index, (**b**) Simpson index, (**c**) Richness index, (**d**) ACE index and (**e**) Chao 1 index were similar between CAP patients with COPD or without COPD. PCA (**f**) and PCoA (**g**) plot were presented as well

Microbiome diversity was compared between CAP with COPD group and CAP without COPD group. Index of shannon, simpson, Richness, ACE, Chao1 were all similar between groups at genus or species level (Fig. 2a-e). Beta diversity was also similar between groups (Fig. 2f-g).

### Differential taxa between groups

**Fig. 3:**
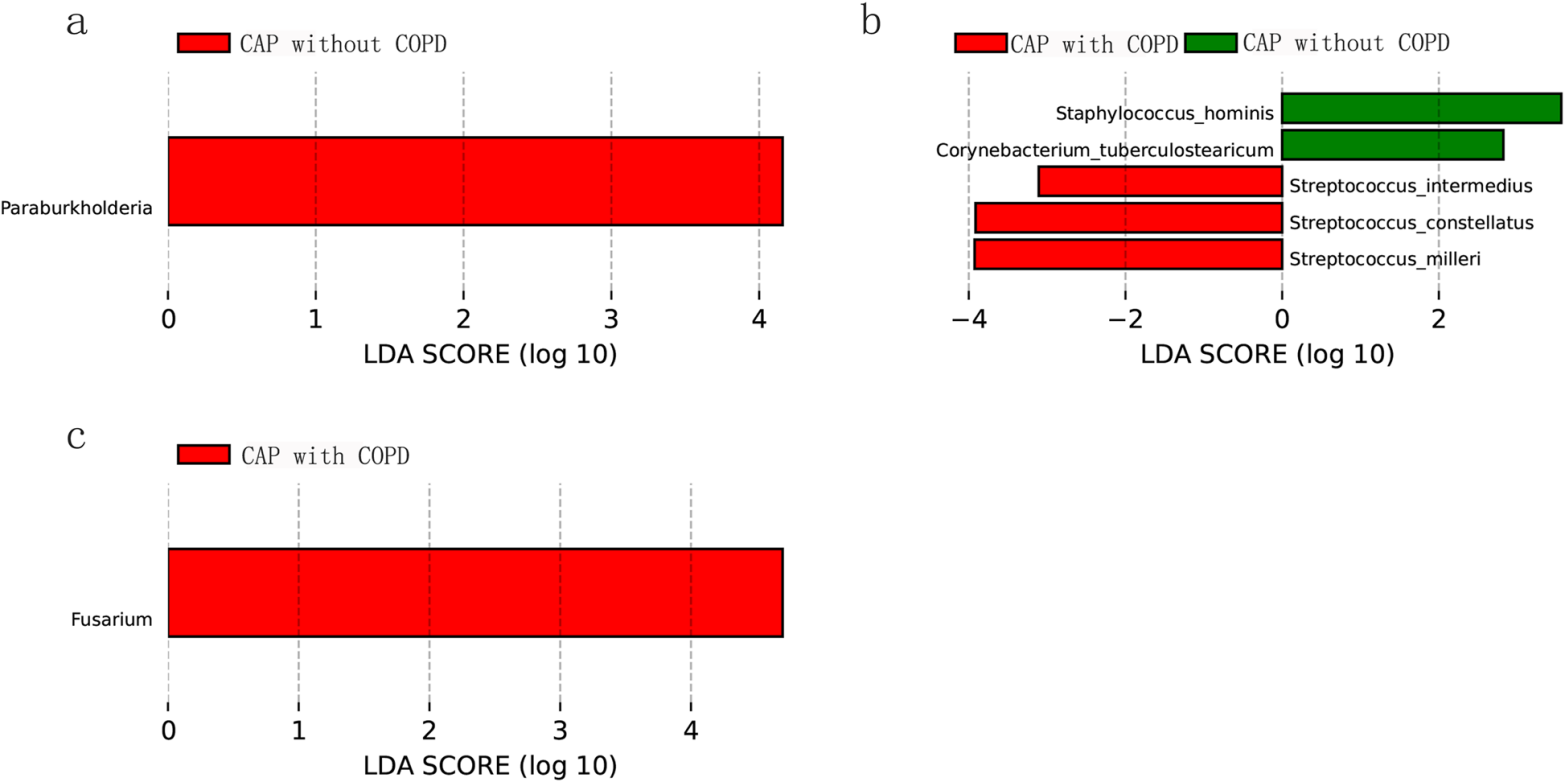
Linear discriminant analysis revealed differentially abundant bacterial taxa of BALF between CAP patients with COPD or without COPD. **a** Differences in bacterial genera between CAP patients with COPD or without COPD. **b** Differences in bacterial species between CAP patients with COPD or without COPD. **c** Difference in fungal and viral genera between CAP patients with COPD or without COPD

A LEfSe analysis was applied to figure out specific microbiome whose abundance were statistically different between CAP without COPD and CAP with COPD group. As shown in the Fig. 3, Paraburkholderia, Corynebacterium tuberculostearicum and Staphylococcus hominis were more enriched in CAP without COPD group while the abundance of Streptococcus intermedius, Streptococcus constellatus, Streptococcus milleri, Fusarium was higher in CAP with COPD group.

### Correlation between the microbiome and clinical indexes

**Fig. 4:**
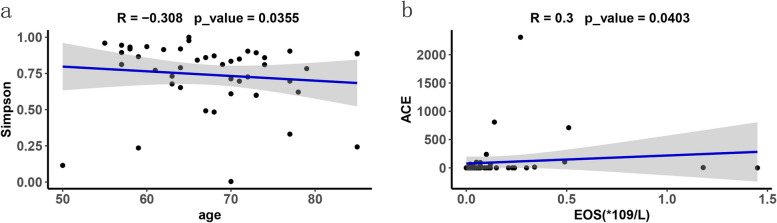
Correlation between BALF microbiome diversity and clinical variables in CAP patients. **a** Age had a negative correlation with Simpson index(*r* = -0.308, *p* = 0.0355). **b** Number of eosinophils had a positive correlation with ACE index (*r* = 0.3, *p* = 0.0403)

The correlation of microbiome diversity index (Richness, ACE, Chao1, Shannon, Simpson, goods Coverage) and clinical indexes (age, smoking index, BMI, PH, PCO_2_, PO_2_, SaO_2_, white blood cell, neutrophils %, leukomonocyte %, eosinophil, CRP, PCT) was analysed. Figure 4 showed that Simpson index was negatively correlated with age (R = -0.308, *p* = 0.0355) while ACE was positively correlated with number of eosinophils (R = 0.3, *p* = 0.0403).

**Fig. 5:**
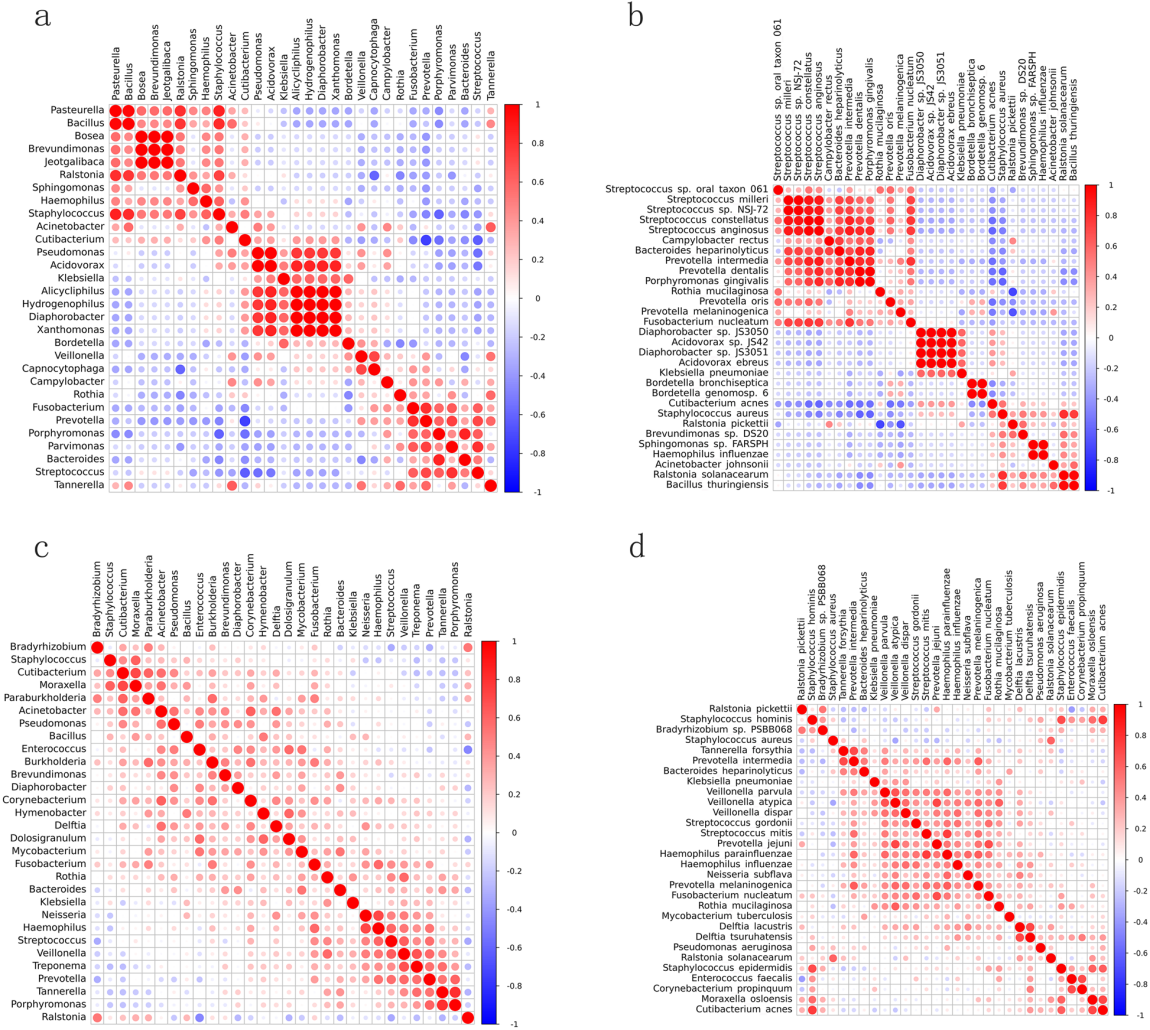
BALF microbiome networks separately in CAP patients with or without COPD. **a** Heatmap of Spearman correlation of top 30 bacterial genera in CAP patients with COPD. **b** Heatmap of Spearman correlation of top 30 bacterial species in CAP patients with COPD. **c** Heatmap of Spearman correlation of top 30 bacterial genera in CAP patients without COPD. **d** Heatmap of Spearman correlation of top 30 bacterial species in CAP patients without COPD

Figure 5 revealed potential co-existence and co-exclusion relationship of top 30 bacteria speices and genus separately in each group.

## Discussion

Clinical pneumonologists tend to make a different empirical antibiotic therapy when faced with a CAP patient concomitant with COPD compared a CAP patient without COPD, considering different pathogenic bacteria tendency. However, no one has looked into the microbiome distribution difference of the two group of patients in detail. This is the first article using mNGS techonology to investigate the BALF microbiome distribution of CAP with COPD and CAP without COPD. We found out that 1) there were no statistical difference in microbiome diversity between CAP with COPD and CAP patients without COPD. 2) The specific distribution of microbiome genera and species were different, including the top 10 bacteria taxa, and the abundance of marginal specific microbiome species were different between groups.

The human respiratory system is inhabited by a diverse microbial community. Our study found that the microbial diversity of BALF was similar between CAP patients with COPD and CAP patients without COPD. In our study, we used several different indexes of diversity including shannon, simpson, Richness, ACE, Chao1, each providing different insights into the richness and evenness of microbial communities. The Shannon index measures both richness and evenness within a community. The Simpson index quantifies diversity by considering the probability that two individuals randomly selected from a sample belong to the same species. The ACE index estimates the total number of species present in a community based on abundance data. It takes into account both observed species richness and the number of rare or singleton species that may not have been observed. Chao1 Index predicts the total number of species in a community by considering the number of rare species observed. Different indexes have different calculation method and different emphasis. Qiang Xiao etc. found that compared with healthy individuals, the bacterial alpha diversity in the lower respiratory tract was significantly lower in CAP patients by 16S rDNA gene sequencing of BALF [23]. Loss of diversity has also been observed in COPD, IPF (idiopathic pulmonary fibrosis) and CF (cystic fibrosis), and it has also been described during exacerbations of these diseases [24].

The LDA analysis identified several different bacteria taxa in each group. It is noteworthy that the three bacterial species which had higher abundance in CAP with COPD group: Streptococcus intermedius, Streptococcus constellatus and Streptococcus milleri are subgroups of viridans streptococci. Viridans streptococci are normal inhabitants of mucous membrane-lined cavities of the animals and humans. They are usually found in the upper respiratory tract, all the regions of the gastrointestinal tract, the female genital tract and mostly in the oral cavity. To be more specific, Inhalation of streptococcus intermedius bacteria in the oropharynx may lead to aspiration pneumonia, and may be complicated with lung abscess and empyema. Streptococcus constellatus was associated with abscess formation in the upper body and respiratory tract. It has also been found to be involved with pulmonary exacerbations in cystic fibrosis patients and can lead to toxic shock and limb amputation [25]. As for the coexistence and coexclusion associations observed in the top 30 specific taxa, there is a possibility that needs to be considered that it could happen also in healthy subjects or COPD patients without pneumonia.

Our study explored the lower airway microbiome of CAP with or without COPD deeply by using mNGS. mNGS is an advanced technology in discovering microbiome, including bacteria, eukaryotes and DNA viruses. Meanwhile mNGS allows unbiased detection of microbiome. Furthermore, mNGS is less influenced by antibiotics compared with traditional culture. Due to limited technology, previous microbiome study could only reveal the microbiome at phylum, class or genus level. But mNGS provide us a higher taxonomic resolution at species level [26–28]. What’s more, we chose BALF sample to reveal the lower airway microbiome, which is also the closest result to those for the bronchial mucosa [29].

There are some limitations for the study. The study was single centered with small number of enrolled patients. Multi-centered clinical trial is needed in the future. Meanwhile, this is a retrospective study of BALF sample. As the bronchoalveolar liquid is mostly diluted in saline, the effect of dilution can have the impact on the result. Although, all the bronchoscope operator is trained to a standard BALF collection procedure. But there may still be some discrepancies during actual operation.

## Conclusions

Although difference of microbiome diversity was no detected between CAP patients with COPD and without COPD. Specific microbiome taxa abundance alteration revealed that concomitant COPD have a mild impact on lower airway microbiome of CAP patients.

## Data Availability

Availability of data and materials Raw sequence data have been deposited in the NCBI “Sequence Read Archive” (SRA) under the project accession PRJNA946338. Other data generated or analyzed during this study are included in this article.

## Abbreviations

CAP: Community-acquired pneumonia
COPD: Chronic obstructive pulmonary dis-ease
mNGS: Metagenomic next generation sequencing
BALF: Bronchoalveolar lavage fluid
LEfSe: Linear discriminant analysis effect size
LDA: Linear discriminant analysis
BMI: Body mass index
